# Validity of three accelerometers to investigate lying, sitting, standing and walking

**DOI:** 10.1371/journal.pone.0217545

**Published:** 2019-05-23

**Authors:** Karin Valkenet, Cindy Veenhof

**Affiliations:** 1 University Medical Center Utrecht, Utrecht University, Department of Rehabilitation, Physical Therapy Science & Sports, The Netherlands; 2 University of Applied Sciences Utrecht, Expertise Center Innovation of Care, Research Group Innovation of Mobility Care, The Netherlands; Indiana University, UNITED STATES

## Abstract

**Background:**

Hospital stays are associated with high levels of sedentary behavior and physical inactivity. To objectively investigate physical behavior of hospitalized patients, these is a need for valid measurement instruments. The aim of this study was to assess the criterion validity of three accelerometers to measure lying, sitting, standing and walking.

**Methods:**

This cross-sectional study was performed in a university hospital. Participants carried out several mobility tasks according to a structured protocol while wearing three accelerometers (ActiGraph GT9X Link, Activ8 Professional and Dynaport MoveMonitor). The participants were guided through the protocol by a test leader and were recorded on video to serve as reference. Sensitivity, specificity, positive predictive values (PPV) and negative predictive values (NPV) were determined for the categories lying, sitting, standing and walking.

**Results:**

In total 12 subjects were included with a mean age of 49.5 (SD 21.5) years and a mean body mass index of 23.8 kg/m^2^ (SD 2.4). The ActiGraph GT9X Link showed an excellent sensitivity (90%) and PPV (98%) for walking, but a poor sensitivity for sitting and standing (57% and 53%), and a poor PPV (43%) for sitting. The Activ8 Professional showed an excellent sensitivity for sitting and walking (95% and 93%), excellent PPV (98%) for walking, but no sensitivity (0%) and PPV (0%) for lying. The Dynaport MoveMonitor showed an excellent sensitivity for sitting (94%), excellent PPV for lying and walking (100% and 99%), but a poor sensitivity (13%) and PPV (19%) for standing.

**Conclusions:**

The validity outcomes for the categories lying, sitting, standing and walking vary between the investigated accelerometers. All three accelerometers scored good to excellent in identifying walking. None of the accelerometers were able to identify all categories validly.

## Introduction

Physical inactivity seems a worldwide epidemic [1;2]. More and more time is spend sitting while prolonged sitting is a risk factor for all-cause mortality, independent of physical activity [3;4]. Whilst this is a recognized problem in daily life, physical inactivity is also a problem within hospital settings [5;6]. The culture in hospitals is to reflexively put patients in their pyjamas in a bed once they are admitted [7–10]. As a result hospitalized patients currently spend 70–83% of the day lying in bed and less than 6% physically active [11–14]. This raises concerns as the dangers of bed rest are well known since decades [5–9].

Interventions that aim to increase inpatient physical activity generally focus at decreasing time spend lying in bed and at increasing time spend sitting, and walking short distances [15;16]. To investigate the effect of these interventions, there is a need for research instruments like accelerometers. To quantify the amount of physical activity of hospitalized patients, the ability of accelerometers to discriminate between lying, sitting, standing and walking is an important requirement. However, the validity of accelerometers to determine these activities is variable [17;18]. Furthermore, since new accelerometers are developed constantly, ongoing research on this topic is required. Three accelerometers that register lying, sitting, standing and walking are the ActiGraph GT9X Link, the Dynaport MoveMonitor and the Activ8 professional. Although there are study reports on the validity of these accelerometers, they have not been validated in for use in a hospital setting.[19–23]

Therefore, the aim of this cross-sectional study is to investigate the criterion validity of the ActiGraph GT9X Link, the Dynaport MoveMonitor and the Activ8 professional to identify lying, sitting, standing and walking.

## Materials and methods

A cross-sectional study was performed in a Dutch university hospital. Participants were eligible for inclusion when they stated that they were able to perform all tasks of a standardized protocol and that they were able to be physically active at moderate intensity for at least 30 consecutive minutes. The study protocol was approved by the medical ethical committee of the University Medical Center Utrecht. Written informed consent was obtained from all participants prior to the assessments.

### Video protocol

The participants wore three accelerometers simultaneously while performing a series of consecutive tasks with a fixed order, according to a continuous 27-minute protocol [24]. The tasks consisted of different body positions and physical activities that are commonly performed during a hospital admission (Table 1). The Activ8 accelerometer registers lying/non-wear only after an absence of movement for a successive period of more than 5 minutes. One of the lying tasks was therefore performed for 6 minutes. All other tasks were performed for 70 seconds. Participants were guided through the protocol by a test leader and were recorded on video (Olympus OM-D E-M10) by a test assistant to serve as reference.

**Table 1:** Structured video protocol and definitions of agreement per accelerometer.

Structured video protocol		Definitions of agreement		
Task	Duration	Activ8	Dynaport	Actigraph*
Lying, in bed in supine position	70 sec	Lying	Lying	Lying
Sitting, on bedside	70 sec	Sitting	Sitting	Sitting
Standing (1)	70 sec	Standing	Standing	Standing and VM = 0
Walking, slow	70 sec	Walking	Shuffling/ Locomotion	Standing and VM>0
Standing (2)	70 sec	Standing	Standing	Standing and VM = 0
Sitting, in stationary chair	70 sec	Sitting	Sitting	Sitting
Walking, fast	70 sec	Walking	Locomotion	Standing and VM>0
Lying, in supine position in transferred bed	6 min	Lying	Lying	Lying
Standing and walking, alternated	70 sec	Standing/ Walking	Shuffling/ Locomotion/ Standing	Standing and VM ≥0
Climbing stairs, 5 steps up and down repeatedly	70 sec	Standing/ Walking	Shuffling/ Locomotion/ Standing	Standing and VM ≥0
Walking, on a treadmill (1 km/h)	70 sec	Walking	Shuffling/ Locomotion	Standing and VM >0
Walking, on a treadmill (2 km/h)	70 sec	Walking	Locomotion	Standing and vector >0
Walking, on a treadmill (3 km/h)	70 sec	Walking	Locomotion	Standing and VM >0
Walking, on a treadmill (4 km/h)	70 sec	Walking	Locomotion	Standing and VM >0
Lying, in bed in lateral position	70 sec	Lying	Lying	Lying
Sitting, in transferred wheelchair	70 sec	Sitting	Sitting	Sitting
Cycling, on ergometer	70 sec	Cycling	NA	NA
Walking, with an infusion pole	70 sec	Walking	Shuffling/ Locomotion	Standing and VM >0
Walking, with a walker rollator	70 sec	Walking	Shuffling/ Locomotion	Standing and VM >0
**Total time**	**27 min**			

*VM: Vector Magnitude (counts per second). A VM of 0 implies no movement, whereas an increasing VM linearly corresponds with increasing movement intensity. Km/h = kilometers per hour (1 kilometer equals 0.62 miles); NA = Not Applicable

### Accelerometers

The ActiGraph GT9X Link (ActiGraph LLP, Pensacola, FL, USA), the Activ8 Professional (Activ8, Valkenswaard, The Netherlands) and the Dynaport MoveMonitor (McRoberts, The Hague, The Netherlands) were used. These 3-axial accelerometers are capable of discriminating between different body postures and physical activities.[19;21;23] Specifications of the accelerometers are displayed in Table 2. The accelerometers were used according to their intended use, no changes in the algorithms were made.

**Table 2:** Specifications of the accelerometers.

Activity monitor	Dimensions, weight, battery duration*	Sensors	Wearing position	Registered body postures and activities	Additional measures	Feedback for commercial use	Data format for professional use (sample rate; interval data output)	Costs**
**ActiGraph GT9X Link**	35x35x10mm, 14 grams, up to 14 days	3-axial accelerometer, gyroscope, magnetometer, secondary accelerometer	Hip	Lying, sitting, standing	Energy expenditure, steps, sleep time, raw acceleration	Programmable LCD display and mobile application	.csv file (30 to 100 Hertz; counts per 1 second)	Hardware: ++ Software: +++
**Activ8 Professional**	30x32x10mm, 20 grams, up to 30 days	3-axial accelerometer	Upper thigh	Lying/non-wear, sitting, standing, walking, cycling, running	Energy expenditure	Emoticon feedback on device	.csv file (12.5 Hertz; counts or MET per 5 minutes, 1 minute or 5 seconds)	Hardware: + Software: free
**Dynaport MoveMonitor**	107x58x12m, 55 grams, up to 14 days	3-axial accelerometer, magnetometer	Lower back	Lying, sitting, standing, shuffling, locomotion	Energy expenditure, steps, sleep movements	Structured pdf reports per 24 hours (to be requested online)	.csv and/or .Rda, .mat, .txt file (50, 100, 200 Hertz; counts per 1 minute and at posture change)	Hardware:+++ Software: +

Dimensions = Length x Width x Thickness, Shuffling = slow walking without registering steps

* depending on of the data output interval

** per accelerometer: + = 1–200 euro, ++ = 201–500 euro, +++ = > 500 euro

The **ActiGraph** was clipped to the participants’ trouser at the hip. Besides lying, sitting and standing, this accelerometer registers a vector magnitude (VM) representing the intensity of the movement. A VM of 0 implies no movement, whereas an increasing VM linearly corresponds with increasing movement intensity. Since the ActiGraph does not register walking, walking was arbitrarily defined as standing in combination with a VM higher than 0. Standing was defined as standing in combination with a VM equal to 0. The interval of the data sampling was 1 measurement per second. The **Activ8** was attached with a plaster at the front of the upper thigh and the lowest interval for the data sampling was used (i.e. 1 measurement per 5 seconds). The **Dynaport** was worn on the lumbar waist using an elastic band. The interval of the data sampling was 1 measurement per second.

### Data analyses

Per participant the performed tasks that were recorded on video were listed per 1 second in a data file. The data from the accelerometers were also listed per 1 second and subsequently were synchronized manually with the data of the video recordings. Cross tables per accelerometer were created to present the registered accelerometer output during each task of the protocol. Sensitivity values were determined per task. Following, the sensitivity, specificity, positive predictive values (PPV) and negative predictive values (NPV) were calculated for the categories lying, sitting, standing and walking. Cycling, stair climbing and alternating standing/walking were not included in these analyses. The analyses per category were corrected for the fact that the task ‘Lying, in supine position in transferred bed’ occurred longer (360 sec instead of 70 sec) compared to the other tasks. Data during the transition from one body position to another were omitted from all analyses. To rank the outcomes, the following four levels were used: 0–70% weak, 71–80% moderate, 81–90% good and 91–100% excellent [25].

## Results

Between April and July 2017, 12 subjects (9 male / 3 female) completed the structured protocol. Of them 4 were healthy subjects, 6 were outpatient subjects and 2 were inpatient subjects. The participants had a mean age of 49.5 (SD 21.5) years and a mean body mass index of 23.8 (SD 2.4) kg/m^2^.

The **ActiGraph** showed good to excellent sensitivity, specificity, PPV and NPV for walking, but poor to moderate sensitivity and PPV for sitting and standing (Tables 3 and 4). During sitting, the ActiGraph registered lying or standing in 27% and 14% of the observations respectively (S1 Table). The scores for lying ranged from poor to excellent (Tables 3 and 4). The **Activ8** showed excellent sensitivity, specificity, PPV and NPV for walking, but no sensitivity and PPV for lying (Tables 3 and 4). During lying, the Activ8 registered sitting in 95% of the samples (S2 Table). The scores for sitting and standing ranged from poor to excellent (Table 4).

**Table 3:** Sensitivity per accelerometer, per task.

Tasks video protocol	Sensitivity*		
	ActiGraph	Activ8	Dynaport
Lying, in bed in supine position	74%	0%	82%
Lying, in supine position in transferred bed	91%	0%	100%
Lying, in bed in lateral position	71%	0%	50%
***Lying*, *average***	***79%***	***0%***	***77%***
Sitting, in stationary chair	66%	100%	89%
Sitting, on bedside	45%	92%	100%
Sitting, in transferred wheelchair	60%	87%	93%
***Sitting*, *average***	***57%***	***93%***	***94%***
Standing (1)	55%	76%	16%
Standing (2)	50%	82%	10%
***Standing*, *average***	***53%***	***79%***	***13%***
Walking, fast	100%	81%	100%
Walking, slow	93%	91%	78%
Walking, on a treadmill (1 km/h)	60%	89%	46%
Walking, on a treadmill (2 km/h)	84%	100%	99%
Walking, on a treadmill (3 km/h)	95%	100%	100%
Walking, on a treadmill (4 km/h)	99%	100%	97%
Walking, with an infusion pole	98%	98%	93%
Walking, with a walker rollator	92%	98%	90%
***Walking*, *average***	***90%***	***95%***	***88%***
Standing and walking, alternated	85%	98%	100%
Climbing stairs, 5 steps	97%	100%	100%
Cycling, on ergometer	NA	98%	NA

* poor: 0–70%, moderate: 71–80%, good: 81–90%, excellent: 91–100% ^25^

Km/h = kilometers per hour (1 kilometer equals 0.62 miles); NA = Not Applicable

**Table 4:** Validity* outcomes per accelerometer, per category.

	ActiGraph				Activ8				Dynaport			
	*Sensitivity*	*Specificity*	*PPV*	*NPV*	*Sensitivity*	*Specificity*	*PPV*	*NPV*	*Sensitivity*	*Specificity*	*PPV*	*NPV*
**Lying**	79%	95%	70%	96%	0%	100%	0%	100%	77%	100%	100%	96%
**Sitting**	57%	88%	43%	92%	93%	82%	28%	98%	94%	86%	57%	99%
**Standing**	53%	98%	74%	95%	79%	95%	73%	97%	13%	92%	19%	90%
**Walking**	90%	99%	98%	94%	95%	96%	96%	95%	88%	99%	99%	93%

Analyses have been corrected for the longer task period of the task ‘Lying, in supine position in transferred bed’.

PPV = Positive Predictive Value; NPV = Negative Predictive Value

* poor: 0–70%, moderate: 71–80%, good: 81–90%, excellent: 91–100% ^25^

The **Dynaport** showed excellent sensitivity for sitting, good to excellent specificity, PPV and NPV for walking, moderate to good sensitivity and PPV for lying, but poor sensitivity and PPV for standing (Tables 3 and 4). During standing, the Dynaport registered sitting in 84% of the samples (S3 Table).

## Discussion

The results of this study show that the validity of the ActiGraph GT9X Link, Activ8 Professional and Dynaport MoveMonitor is good to excellent for registering walking. The validity, based on the positive predictive values, of the ActiGraph and Activ8 is poor for lying and sitting, moderate for standing and excellent for walking. The validity of the Dynaport is poor for sitting and standing, and excellent for lying and walking.

The differences in validity outcomes per accelerometer can mainly be explained by the location of the instrument on the body [18]. We followed the manuals of the accelerometers to determine accelerometer placement. The position of an accelerometer worn on the lower back is similar during sitting and standing which results in the same angle of the device in these postures. Furthermore, due to the absence of motion during sitting or standing, the acceleration of the accelerometer is small in both positions. Accelerometers placed on the lower back are therefore less able to discriminate between these two postures. This may explain the poor PPV values of the Dynaport for sitting and standing. For accelerometers worn at the front of the upper thigh the same explanation applies. Its position does not change when transitioning from lying to sitting (or vice versa) resulting in more difficulty in differentiating between these body positions. This may explain the poor PPV values for lying and sitting of the Activ8. Our results show that the hip appears to be the location with the least outliers in the PPV values for the identification of lying, sitting, standing and walking.

Investigating the validity of accelerometers is important as incorrect registration of postures or activities may lead to wrongful interpretation of data. For example, the results show that the Activ8 scored sitting instead of lying in 95% of the observations which may lead to a large overestimation of the amount of time spent sitting. A report on the validity of the Activ8 concluded moderate to good validity in detecting sitting, standing and walking[21]. Lying however was not investigated since their protocol included tasks with a maximum duration of 2 minutes while the Activ8 only registers lying/non-wear after an absence of movement for a successive period of more than 5 minutes [21]. To allow the Activ8 to identify lying in our study, one task consisted of lying in bed for 6 minutes. Nevertheless, lying was never registered by the Activ8 resulting in 0% sensitivity and PPV for this posture. This may have important implications for use in a hospital setting were patients spend most of their time lying [26].

Our results of the Dynaport MoveMonitor are in line with earlier validation studies concluding that the validity for the detection of walking and lying was good to excellent, whereas standing was pre-dominantly less well detected [19;20]. The study of Fokkenrood et al [20] concluded high sensitivity and PPV for sitting of the MoveMonitor while our results showed high sensitivity but poor PPV for sitting.

The inclinometer of the ActiGraph only reports the postures lying, sitting and standing [23]. Standing entails the upright position of the body during both stationary standing and walking.

To differentiate between standing and walking we combined the inclinometer and the accelerometer output [22;23]. We defined standing as standing with a VM of 0 and walking as standing with a VM higher than 0. It could be argued that our definition for standing was fairly strict since the threshold for sedentary behaviors (including standing) is 2.5 counts per second and small movements during standing might lead to increases in VM [22]. Our data however shows that during standing the ActiGraph reported a VM higher than 0 in only 3% of the observations. During walking, the accelerometer recorded a VM higher than 0 in 100% of the cases and the VM linearly increased with increasing walking speeds (S1 Table). Although combining the inclinometer and accelerometer output appears to result in valid data for the differentiation between standing and walking, this method had not been validated.

We stated that the most important requirement of an accelerometer to objectify the amount of physical activity in a hospital setting is its ability to discriminate between lying, sitting, standing and walking as these are the most common body postures and physical activities performed by hospitalized patients. Furthermore, these accelerometers would then allow objective evaluation of initiatives aiming to decrease sedentary behavior in hospitalized patients by shifting the time spend lying in bed to time spend sitting and walking [15;16]. Other outcome measures and parameters can nonetheless be preferred to objectify the amount of physical activity of hospitalized patients. For example, a recent brief report stated that walking fewer than 900 steps per day during acute hospitalization is strongly associated with functional decline [27]. However, the validity per accelerometer of counting steps has to be investigated specifically for use in hospital settings since accelerometers tend to become less valid at lower walking speeds.[20] This also applies for the registration of energy expenditure as the energy expenditure of patients may not be comparable with healthy individuals [28].

A limitation of our study is that standardized protocols lack ecological validity as activities are not performed in a natural way and order. Therefore, assessing the validity of accelerometers during an average (hospital) day is advised in future research [24]. Our study focused on validating the accelerometers during specified body postures and physical activities rather than on detecting movement behavior characteristic of hospitalized patients. Therefore also healthy participants could be included while inclusion of only hospitalized patients might have added value to our report. Finally, although our sample size is comparable to similar studies, a larger sample size would have led to better generalizability of the results.

In conclusion we state that all three accelerometers are able to validly discriminate between certain postures and physical activities, but that there are important differences between the instruments. These differences can have important consequences for the validity of the data output and should therefore be taken into consideration when determining the instrument and outcome measure of choice for research on physical behavior.

## Supporting information

S1 Table Registration of the ActiGraph in comparison with the video protocol. (DOCX)

S2 Table Registration of the Activ8 in comparison with the video protocol. (DOCX)

S3 Table Registration of the Dynaport in comparison with the video protocol. (DOCX)
