# Supplementary material for: Validity of three accelerometers to investigate lying, sitting, standing and walking
Source: PLoS One. 2019 May 23;14(5):e0217545. doi: 10.1371/journal.pone.0217545 (PMC6532937; doi:10.1371/journal.pone.0217545)
Supplement: S1 Table — (DOCX) [file pone.0217545.s001.docx]

**S1 Table. Registration of the ActiGraph in comparison with the video protocol**

| **Video protocol** | **Actigraph registration*** | | | | | | |
| --- | --- | --- | --- | --- | --- | --- | --- |
|  | **Vector (mean)** | **Walking**** | **Standing** | **Sitting** | **Lying** | **Off***** | **Total** |
| **Lying, supine** | 0 | 10 | 0 | 69 | **622** | 141 | 842 |
| **Lying, transferred bed** | 0 | 22 | 1 | 367 | **3860** | 5 | 4255 |
| **Lying, on side** | 3 | 11 | 3 | 82 | **376** | 59 | 531 |
| **Sitting, chair** | 1 | 11 | 63 | **523** | 196 | 0 | 793 |
| **Sitting, bedsite** | 1 | 13 | 189 | **344** | 190 | 34 | 770 |
| **Sitting, transferred chair** | 4 | 24 | 5 | **417** | 228 | 18 | 692 |
| **Standing (1)** | 2 | 32 | **435** | 329 | 0 | 0 | 796 |
| **Standing (2)** | 1 | 17 | **403** | 353 | 29 | 0 | 802 |
| **Walking, fast** | 91 | **827** | 0 | 3 | 0 | 0 | 830 |
| **Walking, slow** | 52 | **791** | 7 | 51 | 2 | 0 | 851 |
| **Walking, treadmill 1 km/h** | 31 | **367** | 20 | 204 | 22 | 0 | 610 |
| **Walking, treadmill 2 km/h** | 43 | **676** | 0 | 114 | 13 | 0 | 803 |
| **Walking, treadmill 3 km/h** | 51 | **784** | 0 | 26 | 17 | 0 | 827 |
| **Walking, treadmill 4 km/h** | 61 | **808** | 0 | 7 | 0 | 0 | 815 |
| **Walking, infusion pole** | 53 | **748** | 0 | 17 | 1 | 0 | 766 |
| **Walking, walker rollator** | 53 | **737** | 1 | 67 | 0 | 0 | 805 |
| **Standing and walking** | 50 | **615** | **56** | 118 | 2 | 0 | 791 |
| **Climbing stairs** | 76 | **769** | **0** | 24 | 0 | 0 | 793 |
| **Cycling** | 66 | 516 | 2 | 126 | 1 | 0 | - |
| **Transfers** | 65 | 1077 | 74 | 209 | 59 | 2 | - |

Marked in yellow and bold: Identical samples of the video recordings and the accelerometer

*Number of observations, unless otherwise stated, ** Walking was determined by combining the inclinometer data (posture=standing) with

the accelerometer data (vector >0), ***Inclinometer indicated that the device was not worn
