# Supplementary material for: Validity of three accelerometers to investigate lying, sitting, standing and walking
Source: PLoS One. 2019 May 23;14(5):e0217545. doi: 10.1371/journal.pone.0217545 (PMC6532937; doi:10.1371/journal.pone.0217545)
Supplement: S2 Table — (DOCX) [file pone.0217545.s002.docx]

**S2 Table. Registration of the Activ8 in comparison with the video protocol**

| **Video protocol** | **Activ8 registration*** | | | | | | | |
| --- | --- | --- | --- | --- | --- | --- | --- | --- |
|  | **Cycling** | **Running** | **Walking** | **Standing** | **Sitting** | **Lying /non-wear** | **Missing **** | **Total** |
| **Lying, supine** | 0 | 2 | 28 | 1 | 742 | **0** | 69 | 773 |
| **Lying, transferred bed** | 0 | 0 | 0 | 0 | 3905 | **0** | 350 | 3905 |
| **Lying, on side** | 0 | 0 | 36 | 193 | 254 | **0** | 48 | 483 |
| **Sitting, chair** | 0 | 0 | 1 | 0 | **725** | 0 | 67 | 726 |
| **Sitting, bedsite** | 0 | 1 | 4 | 0 | **645** | 0 | 69 | 701 |
| **Sitting, transferred chair** | 19 | 0 | 0 | 64 | **545** | 0 | 64 | 628 |
| **Standing (1)** | 0 | 0 | 140 | **555** | 32 | 0 | 69 | 727 |
| **Standing (2)** | 0 | 0 | 19 | **601** | 114 | 0 | 68 | 734 |
| **Walking, fast** | 0 | 140 | **615** | 0 | 2 | 0 | 73 | 757 |
| **Walking, slow** | 0 | 0 | **684** | 65 | 0 | 0 | 102 | 749 |
| **Walking, treadmill 1 km/u** | 0 | 0 | **499** | 62 | 0 | 0 | 49 | 561 |
| **Walking, treadmill 2 km/u** | 0 | 0 | **741** | 0 | 0 | 0 | 62 | 741 |
| **Walking, treadmill 3 km/u** | 0 | 0 | **763** | 0 | 0 | 0 | 64 | 763 |
| **Walking, treadmill 4 km/u** | 0 | 1 | **742** | 0 | 0 | 0 | 72 | 743 |
| **Walking, infusion pole** | 10 | 1 | **688** | 0 | 0 | 0 | 67 | 699 |
| **Walking, walker rollator** | 13 | 3 | **722** | 0 | 0 | 0 | 67 | 738 |
| **Standing and walking** | 0 | 1 | **600** | **94** | 14 | 0 | 82 | 709 |
| **Climbing stairs** | 0 | 0 | **723** | **1** | 0 | 0 | 69 | 724 |
| **Cycling** | **586** | 0 | 13 | 0 | 0 | 0 | 46 | 599 |
| **Transfers** | 80 | 10 | 784 | 78 | 343 | 0 | 128 | - |

Marked in yellow and bold: Identical observations of the video recordings and the accelerometer

*Number of observations, unless otherwise stated., ** Data is missing of 1 participant due wrong settings of the data output.
