# Supplementary material for: Validity of three accelerometers to investigate lying, sitting, standing and walking
Source: PLoS One. 2019 May 23;14(5):e0217545. doi: 10.1371/journal.pone.0217545 (PMC6532937; doi:10.1371/journal.pone.0217545)
Supplement: S3 Table — (DOCX) [file pone.0217545.s003.docx]

**S3 Table. Registration of the Dynaport MoveMonitor in comparison with the video protocol**

| **Video protocol** | **Dynaport registration*** | | | | | | |
| --- | --- | --- | --- | --- | --- | --- | --- |
|  | **Loco motion** | **Shuffling** | **Standing** | **Sitting** | **Lying** | **Missing**** | **Total** |
| **Lying, supine** | 0 | 0 | 0 | 71 | **333** | 438*** | 404 |
| **Lying, transferred bed** | 0 | 0 | 0 | 0 | **3197** | 1058 | 3197 |
| **Lying, on side** | 0 | 0 | 141 | 67 | **209** | 114 | 417 |
| **Sitting, chair** | 0 | 2 | 63 | **528** | 0 | 200 | 593 |
| **Sitting, bedsite** | 0 | 0 | 0 | **579** | 1 | 190 | 580 |
| **Sitting, transferred chair** | 1 | 15 | 21 | **484** | 0 | 171 | 521 |
| **Standing (1)** | 12 | 17 | **95** | 476 | 0 | 196 | 600 |
| **Standing (2)** | 0 | 4 | **61** | 532 | 0 | 205 | 597 |
| **Walking, fast** | **627** | 0 | 0 | 0 | 0 | 203 | 627 |
| **Walking, slow** | **484** | **15** | 67 | 73 | 0 | 212 | 639 |
| **Walking, treadmill 1 km/u** | **94** | **122** | 252 | 0 | 0 | 142 | 468 |
| **Walking, treadmill 2 km/u** | **600** | **2** | 5 | 0 | 0 | 196 | 607 |
| **Walking, treadmill 3 km/u** | **606** | 0 | 0 | 0 | 0 | 221 | 606 |
| **Walking, treadmill 4 km/u** | **593** | 0 | 17 | 0 | 0 | 205 | 610 |
| **Walking, infusion pole** | **542** | **8** | 41 | 0 | 0 | 175 | 591 |
| **Walking, walker rollator** | **542** | **3** | 58 | 0 | 0 | 202 | 603 |
| **Standing and walking** | **464** | **0** | **109** | 0 | 1 | 217 | 574 |
| **Climbing stairs** | **605** | **0** | **8** | 0 | 0 | 180 | 613 |
| **Cycling** | 133 | 136 | 106 | 130 | 0 | 140 | - |
| **Transfers** | 452 | 49 | 161 | 176 | 165 | 420 | - |

Marked in yellow and bold: Identical samples of the video recordings and the accelerometer

*Number of observations, unless otherwise stated

** In 2 participants the Dynaport did not measure correctly and only registered lying. Of 1 participant no data is available.
*** In 5 participants the Dynaport started measuring 30 seconds too late resulting in additional missing data on the first task (lying, supine)
